# Cooperativity and Steep Voltage Dependence in a Bacterial Channel

**DOI:** 10.3390/ijms20184501

**Published:** 2019-09-11

**Authors:** Shang H. Lin, Kai-Ti Chang, Nuval Cherian, Benjamin Wu, Hyo Phee, Christy Cho, Marco Colombini

**Affiliations:** Department of Biology, University of Maryland, College Park, MD 20742, USA

**Keywords:** *Escherichia coli*, planar membrane, pore, dipole, electrophysiology, voltage gating

## Abstract

This paper reports on the discovery of a novel three-membrane channel unit exhibiting very steep voltage dependence and strong cooperative behavior. It was reconstituted into planar phospholipid membranes formed by the monolayer method and studied under voltage-clamp conditions. The behavior of the novel channel-former, isolated from *Escherichia coli*, is consistent with a linearly organized three-channel unit displaying steep voltage-gating (a minimum of 14 charges in the voltage sensor) that rivals that of channels in mammalian excitable membranes. The channels also display strong cooperativity in that closure of the first channel permits the second to close and closure of the second channel permits closure of the third. All three have virtually the same conductance and selectivity, and yet the first and third close at positive potentials whereas the second closes at negative potentials. Thus, is it likely that the second channel-former is oriented in the membrane in a direction opposite to that of the other two. This novel structure is named “triplin.” The extraordinary behavior of triplin indicates that it must have important and as yet undefined physiological roles.

## 1. Introduction

Voltage-gated membrane channels have evolved to be especially sensitive to changes in membrane potential. To achieve this, these channels possess a voltage sensor whose movement is coupled to the conformational state of the protein and in this way determine the functional state of the channel (closed or open). Voltage-gated channels are most often studied in cells possessing electrical excitability (e.g., muscles and neurons) [1,2]. In these systems, the steep voltage dependence underlies the ability of these cells to generate and propagate action potentials and a variety of electrical phenomena. In addition to these, voltage-gated channels are important in the function of a wide variety of cells (e.g., osteoblasts [3], endocrine cells [4], dynoflagellates [5], and paramecia [6]). Voltage gated channels also exist in prokaryotes. Those studied to date are members of the Na^+^/K^+^/Ca^++^ channel family and homologous to mammalian channels [7,8]. These are highly selective for small ions. Another family of channels exists in prokaryotes. These are collectively referred to as porins and are located in the outer membrane. Some show voltage dependence, but it is very weak (low effective gating charge) [9], and the physiological role of this voltage dependence has been tested and found lacking [10]. Here, we report the discovery of membrane channels with pore sizes similar to those of the major porins but having very steep voltage dependence, quite similar to that of the small ion channels. We have named this new channel-former “triplin.” Steep voltage dependence is synonymous with sophisticated regulation of major functions involving the entire cell. These are well known in mammalian cells but are very poorly understood in prokaryotes. An exception is the recent publication [11], which shows how voltage gated calcium ion channels in *Escherichia coli* mediate mechanosensation. Excitable phenomena in bacteria were also reported [12]. Thus, the discovery of steep voltage dependence in the larger pore-formers, indicates the presence of as yet not understood critical functional systems.

## 2. Results

Soluble fractions from *E. coli* were purified using a chitin column, and the bound material was eluted with dithiothreitol (DTT). The DTT was dialyzed away. Channel formation in planar phospholipid membranes [13,14] was observed when this material was treated with octyl glucoside and dispersed into the aqueous solution bathing the membrane. These membrane channels have properties unlike any described before, including very steep voltage dependence and high cooperativity.

### 2.1. Formation of Novel Channels

**Figure 1:**
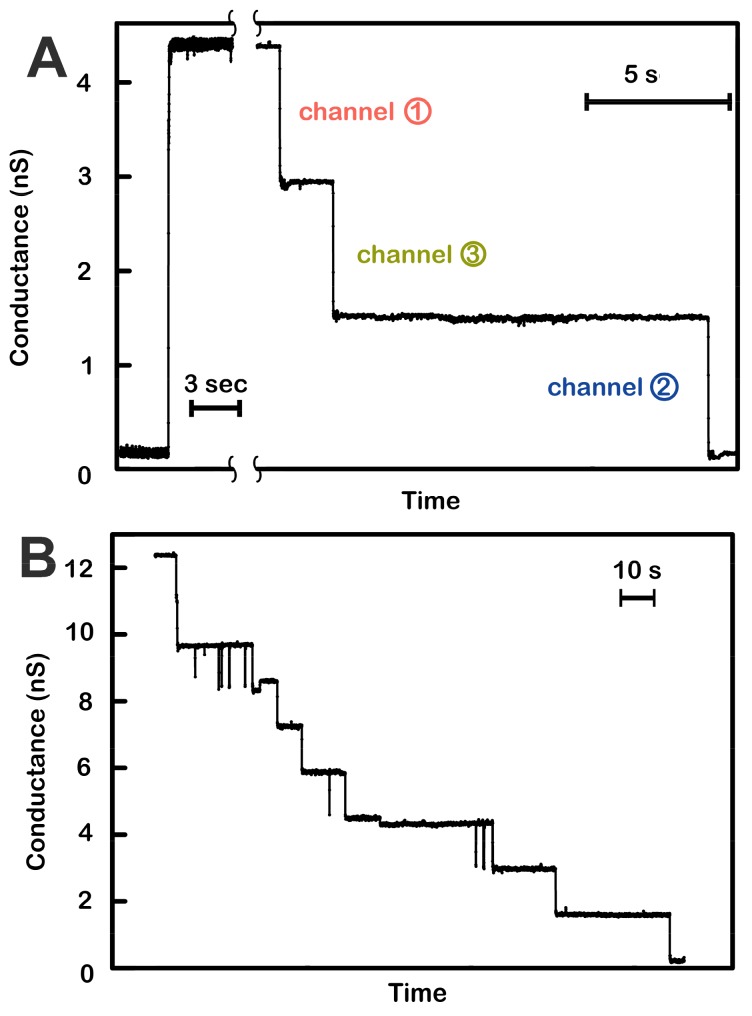
Voltage-dependent channel closure of triplins inserted into planar phospholipid membranes. (**A**) Insertion of a single triplin into a planar phospholipid membrane. The single insertion event (left) took place under a +10 mV applied potential. When the potential was raised to +70 mV, three conductance drops took place (right). The indicated order of closure (channel 1, 3, then 2) is based on the model presented in Figure 7. (**B**) A +70 mV transmembrane potential was applied to a planar membrane containing three triplins. Nine conductance decrements took place, equivalent to the closure of all the channels formed by the three triplins.

The only observed conductance with clear characteristics distinguishing it from non-specific conductances is that due to the novel channel-former named triplin, whose characteristics will be described below. Channels with these properties were observed in more than 100 experiments performed on samples from more than 30 separate isolations. The functional unit has a conductance of 4.4 ± 0.1 nanoSiemens (nS) (19 separate experiments) in 1.0 M KCl, and the number of conductance units that form following the dispersal of the sample varied from one experiment to another. The properties of triplin are best seen when a single entity is present in the membrane (Figure 1A). Although the insertion was a single event, increasing the applied voltage from +10 to +70mV resulted in three distinct conductance drops of essentially equal magnitude. Figure 1B shows the closing events recorded with a membrane containing three triplins. Note the very slow rate of channel closure. This is in very sharp contrast with that seen using voltage ramps (below). These channel closures were not observed when a negative potential was applied (Figure S1), indicating both an asymmetrical gating behavior and a unidirectional insertion process.

**Figure 2:**
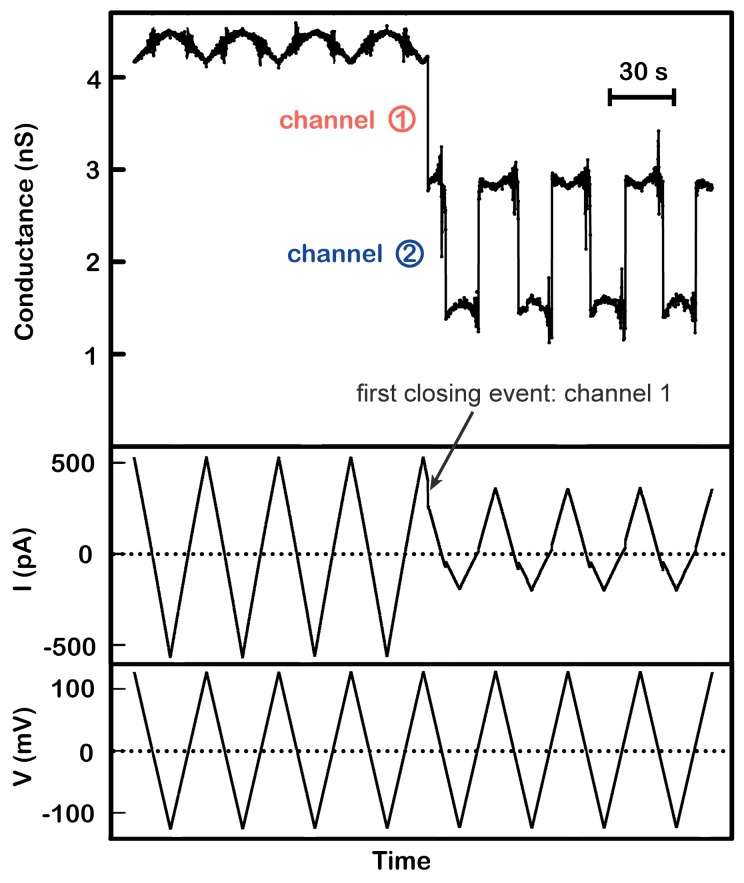
Positive cooperativity between channel 1 and 2. A single triplin (4.4 nS) was probed using a 30 mHz (7.4 mV/s) triangular voltage ramp (±124 mV). From top to bottom, the panels are the calculated conductance, the current (in picoAmperes, pA), and the applied voltage traces. Closure of channel 2 at negative potentials only occurred after closure of channel 1 at a high positive potential (here at +98 mV). Channel 2 closure and reopening occurred in all subsequent voltage ramps. Note that in the absence of gating the conductance varies somewhat with voltage due to a small amount of rectification in these channels.

The three distinct closing events demonstrate that the triplin behaves as if it was composed of a triplet of channels. Accepting this interpretation, the channels are designated as channel 1, 2, and 3 because they have distinct properties. To introduce these properties, a representative experiment is shown in Figure 2.

**Figure 3:**
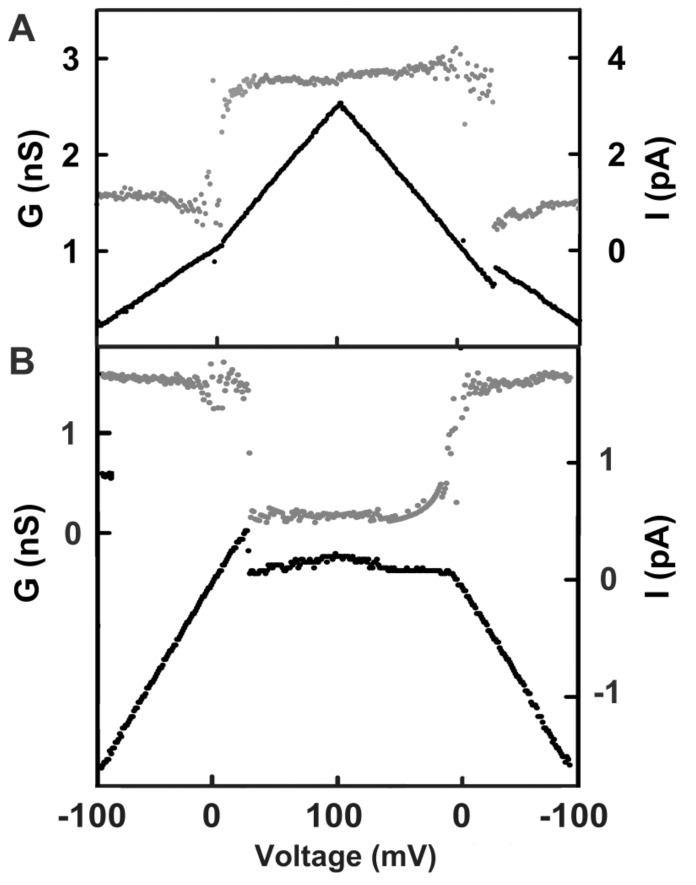
Gating pattern of channels 2 and 3. The membrane contained a single triplin, and by the time these segments were recorded, channel 1 had closed. Illustrated in each panel is the current recorded (lower trace, using the scale at the right) during one cycle of a triangular voltage ramp (31 mHz, ± 100 mV). The upper trace (gray dots) is the calculated conductance, using the scale at the left. (**A**) Opening and closing of channel 2. Note that channel 2 closure took place at negative potentials, whereas the reopening, being kinetically delayed, occurred at a slightly positive voltage. (**B**) Closing and opening of channel 3 when channels 1 and 2 remained closed. Channel 3 closed at positive voltages, and the remaining conductance was close to zero.

The application of 7.4 mV/s voltage ramps to a single triplin (4.4 nS in this case) shows the total absence of voltage gating for several cycles. When all channels are open they display some form of cooperative stabilization resulting in no gating. However, a conductance drop of 1.5 nS at a high positive voltage (closure of channel 1) resulted in gating at negative voltages in all subsequent ramp cycles performed in this experiment (90 in this experiment; Figure 2 only shows the first 4). This is characteristic behavior. In all experiments, voltage gating is initiated (permitted?) only after a first closing event, defined as closure of channel 1. The first conductance drop always occurs at a high positive potential (+98 mV for the example shown in Figure 2 but typically taking place at anywhere above +70mV). A high positive potential is always needed to activate the voltage-gating process, and this was generally done by applying a constant voltage of +70 to +100 mV depending on the experiment. The subsequent gating that took place at such regular intervals was due to the closure and reopening of channel 2. Closure of channel 2 took place at negative voltages. Boltzmann analysis resulted in a voltage that closed half the channels (V_0_) to be −27 ± 4 mV, when using 7.4 mV/s ramps (for 70 separate experiments done with 4.5 mV/s ramps the average value was −31 ± 2 mV). Note that the closure of channel 2 occurred at electrical potentials of a sign opposite to that required for channel 1 closure indicating that the gating charges in these two channels are moving in the opposite direction. Alternatively, the gating charges would have to be of opposite sign. Channel 2 reopening took place as the potential became more positive. This can be seen more clearly in Figure 3A, where a single voltage cycle from another experiment is shown in an expanded view. 

**Figure 4:**
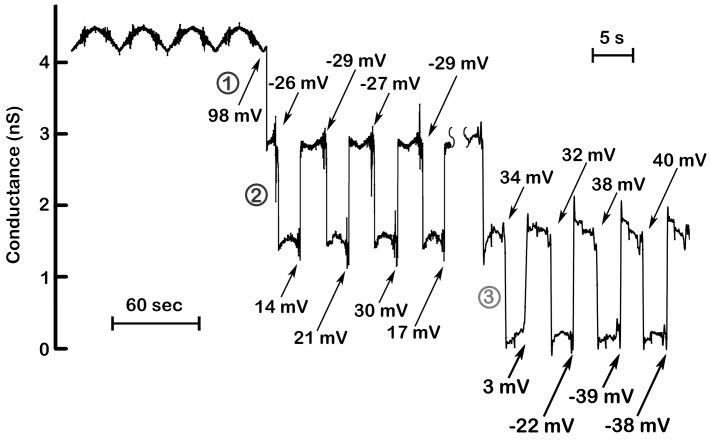
Steep voltage dependence and cooperativity of channel 2 and 3. The results illustrated in Figure 2 were supplemented with those collected at a later time in the same experiment but recorded while applying triangular ramps at a higher frequency (175 mHz; 43.4 mV/s). The numbers indicate the voltages at which the channels closed or opened. Channel 2 closed at nearly identical voltages (−26, −29, −27, −29 mV), whereas reopening was delayed to positive voltages due to slower kinetics of channel reopening. (Note the wide variation in the voltages at which channel 2 reopened) At higher ramp frequencies (right), the slow reopening kinetics of channel 2 resulted in its remaining closed at the positive potentials needed to close channel 3. Again, note that the voltages at which channel 3 closed are in a narrow range whereas the voltages at which opening occurred varied widely.

In the experiment illustrated in Figure 2, the gating caused the conductance to shift between 2.9 and 1.5 nS consistent with gating of only channel 2. In all experiments, channel 1 reopening was very rare, and if it took place, all gating was stopped until a high positive potential was again used to close channel 1. The average value of the conductance drop due to the closure of channel 2 was 1.42 ± 0.15 nS (42 experiments). The closure of channel 2 not only occurred at each subsequent cycle (Figure 2), but it occurred at a very narrow voltage range (from −26 to −29 mV in the four events shown in Figure 4). This is not the case for the reopening voltage that occurred at positive voltages. Both the voltage shift between closure and opening and the large range of voltages at which opening took place can be understood as being the result of the slow opening kinetics of these channels compared to rate of voltage change of the applied voltage ramp.

Channel 3 closure occurred at positive potentials with the V_0_ of +29 ± 3 mV (11 experiments) and reopening occurred as the positive potential declined (Figure 3B). The closure of channel 3 occurs only when channel 2 is closed. Since channel 2 reopens as the transmembrane potential turns positive, channel 3 closure should be very rare under these conditions. However, whereas channel 2 closure is kinetically fast, its reopening is kinetically delayed. Thus, increasing the rate of voltage change results in channel 2 remaining open as the voltage becomes positive allowing channel 3 to close. For example, in 215 ramps run at 7.4 mV/s channel 3 closure was observed only 26% of the time whereas in 67 ramps run at 43 mV/s channel 3 closure was observed 84% of the time.

### 2.2. Steep Voltage Gating of the Triplin Channels

**Figure 5:**
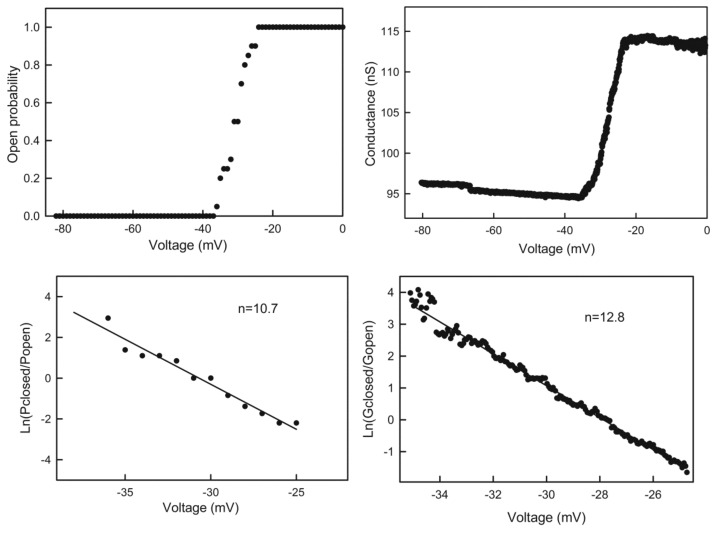
Quantitation of the voltage dependence of triplin. Upper left: the data was collected from an experiment with a single triplin. Twenty consecutive records of the current response to a 30 mHz triangular voltage ramp (±82mV) were analyzed to determine the probability of finding channel 2 open as the voltage declined from 0 to −82 mV. This is the channel closing process. This data was log transformed as described in Methods to yield the plot in the lower left panel. The line is the least squares fit through the data. From the slope of the line, the voltage dependence parameters n and V_0_ were determined, and the n values are indicated. The right panels are similar except that in this experiment the membrane contained many triplins (perhaps 28) only some of which were gating. The triangular voltage ramp was run at 9 mHz (±80 mV).

**Figure 6:**
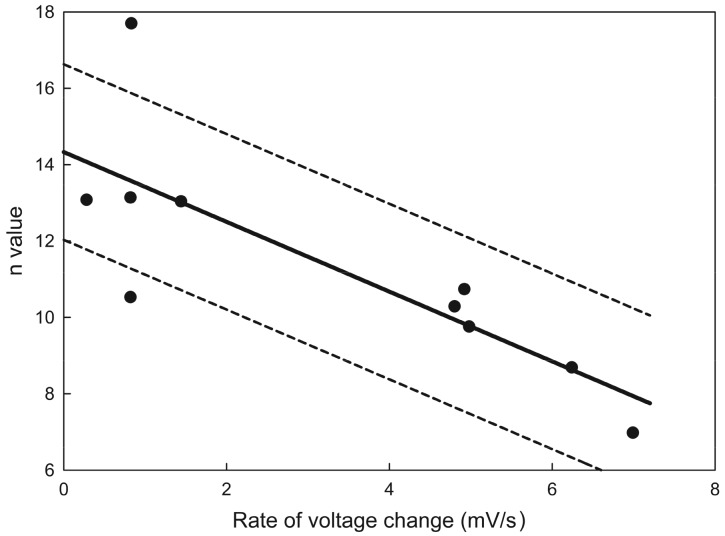
Estimate of the effective number of gating charges, n. The values plotted were the results of Boltzmann distribution fits to the gating of channel 2 as the voltage was varied at different rates. The data originated from ten separate experiments. The solid line is the least squares fit, and the dotted lines show the 95% confidence limits. Extrapolation to zero rate of voltage change yields a value for *n* (equilibrium value) of 14 ± 2.5.

Plots of the probability of a single channel being open as a function of voltage (Figure 5 upper left) or the conductance of a multi-triplin membrane as a function of voltage (Figure 5 upper right) show a steep voltage dependence for the closure of channel 2. The minimum number of charges on the voltage sensor needed to account for this voltage dependence was calculated by fitting to the Boltzmann distribution (see Theory and Calculation section). The rapid kinetics of channel 2 closure permitted the assumption of the achievement of a quasi-equilibrium between the open and closed state as the voltage was changing during the voltage ramp. With this assumption the effective number of gating charges, n, was calculated by fitting to the Boltzmann distribution (Figure 5, lower graphs). These values were found to depend on the rate of change in voltage indicating that the kinetics of the process was still limiting. Thus, the values of n obtained from various experiments were plotted against the rate of voltage change and the value of n at zero rate of voltage change was determined by extrapolation (Figure 6). The resulting n of 14 ± 2 indicates that a minimum of 14 charges would need to translocate through the entire potential difference to account for the observed voltage-dependent gating. This large amount of charge translocation is typical of the channels responsible for the electrical excitability of neurons and muscles.

### 2.3. Kinetic Model Accounts for the Phenomenology

**Figure 7:**
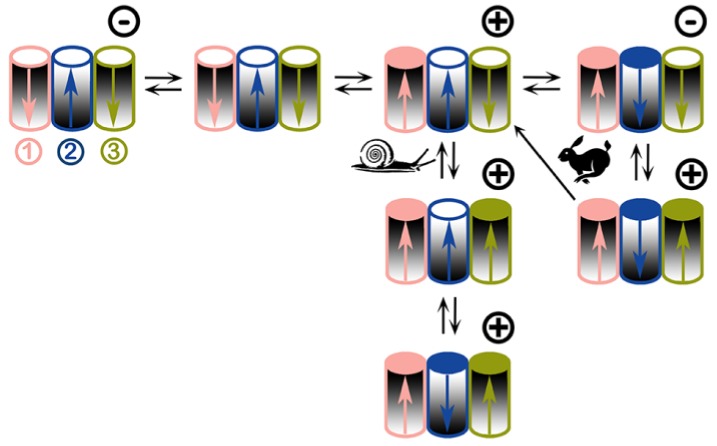
Proposed voltage-gating model of triplin channels. Each triplin consists of three cylindrical channels arranged in a row. The orientation of each channel is indicated by the graded shading. Thus, the middle cylinder (channel 2) has an orientation opposite to that of its neighbors. Each channel has its own gating charge whose asymmetric distribution results in a dipole (arrow). The tip of the arrow is the negative end. An open channel is designated by a white circle on top, a closed one has a colored circle. Channel closure translocates the charged voltage sensor through the membrane thus inverting the dipole moment (i.e. inverting the arrow). The orientation of the channel is not changed. Adjacent channels influence each other through dipole-dipole interactions. The applied voltage is indicated by the symbol at the upper right corner of each triplin. All three channels are open when no potential is applied (upper left). From an all channels open state, a high positive voltage induces closure of channel 1 whereas a negative voltage has no effect. Snail path: with a constant voltage, adjacent dipoles stabilize the structure thus inhibiting channel closure resulting in slow rates of closure. With time a high positive voltage closes both channel 1 and 3. Cooperation with its neighbors forces channel 2 to close despite the wrong sign of the applied voltage. Rabbit path: using triangular voltage ramps, the closure of channel 1 favors closure of channel 2 due to favorable dipole-dipole interaction. Channel 2 closure occurs at negative voltages due to its opposite orientation. Dipole-dipole interaction between 2 and 3 requires 2 to be closed for 3 to close. When fast triangular voltage ramps are applied, the slow rate of opening of channel 2 results in 2 remaining closed at positive potentials long enough for channel 3 to close. This leads to the all-closed structure at the lower right. As the voltage is reduced there are only two outcomes: either channel 3 reopens followed by channel 2 or channel 3 and 2 can reopen simultaneously (one-way arrow).

These results and others presented below can be understood in the context of the model illustrated in Figure 7. This model embodies the experimental observations and gives a mechanistic explanation of the cooperativity and voltage-dependence of triplin. We propose that the fundamental conducting unit of 4.4 nS is a triplet of structurally identical channels in a linear organization. Although a linear organization would be a very unusual structural feature, such an organization would best explain the functional behavior. The closure of channel 1 strongly affects the gating of channel 2 in that it allows channel 2 to close at low transmembrane voltages. Similarly, the closure of channel 2 favors the closure of channel 3. There is no indication that channel 1 influences channel 3 so their dipoles do not seem to interact thus separating these by channel 2 seems appropriate. From the sign of the voltage at which the channels gate, it is proposed that channel 1 and 3 are oriented in one direction and 2 is oriented in the opposite direction (shading direction indicates orientation). The asymmetric charge distribution of the voltage sensor in each channel is indicated by the arrows. This dipolar structure inverts upon voltage-dependent channel closure. Note: this is not a reorientation of the channel itself, just the voltage sensor domain. In the model, the orientation of the voltage sensors was chosen to match the experimental results. Similarly, the model consists of three structurally identical channels because each channel has the same conductance. However, that need not be the case. The behavior described is best explained by channel–channel interactions that produce a strong positive cooperativity favoring the channels to be in the same conformational state, either all open or all closed. The simplest explanation for this cooperativity is to attribute it to dipole–dipole interactions. This naturally ties together the voltage gating and cooperativity rather than proposing two separate processes. The favorable dipole–dipole interaction between channels 1 and 2 make the closure of the first channel (channel 1) unfavored as indicated by the slow rate of closure and the requirement of a high positive potential (Figure 2). When closure does take place, it could be due to the closure of either channel 1 or 3, since these are identical in location and interaction. Once one of these closes, that one is defined as channel 1. Closure of channel 1 and the associated reorientation of the voltage sensor dipole favors the closure of 2. Thus, a much smaller potential is needed to close channel 2 and the kinetics of this process are fast (rabbit symbol in Figure 7). Channel 3 is apparently not affected by channel 1 closure. For channel 3 to close requires both a positive potential (acting on the voltage sensor) and a closed 2 (removing the favorable dipole-dipole interaction). However, a positive potential also leads to channel 2 reopening and therefore the two requirements for channel 3 closure are incompatible. By using a higher rate of voltage change, one takes advantage of the relatively slow reopening kinetics of channel 2 and thus achieve the conditions necessary for the closure of channel 3. Therefore, as the transmembrane electrical potential moves toward positive values, either channel 2 reopens or channel 3 closes. At elevated positive potentials both are either open or closed due to strong cooperativity.

**Figure 8:**
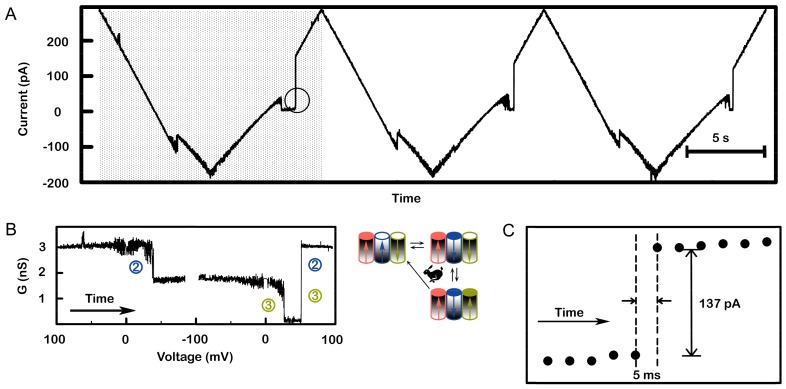
The virtually simultaneous reopening of channel 2 and 3 demonstrates strong cooperative behavior. A single triplin was probed with a 71 mHz (13.6 mV/s) triangular voltage ramp. (**A**) A portion of the recording is shown whereby three successive voltage ramps show essentially simultaneous reopening of channels 2 and 3. (**B**) A conductance vs. voltage plot of the data in the shaded area in (A) was generated and annotated to indicate which channels are gating. Both (A) and (B) show channel 2 closure at a negative voltage, then channel 3 closing as the potential became positive, followed by simultaneous reopening of channels 2 and 3. (**C**) Expanded view of the circled area in (A) showing that the current increased within 5 ms.

**Figure 9:**
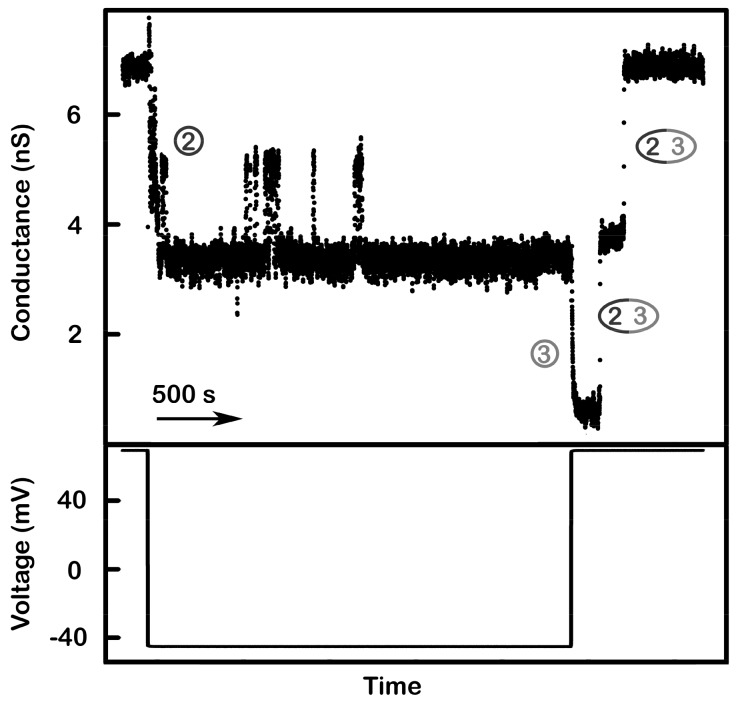
Model tested by voltage pulse sequence. This membrane contained two conducting triplins (total conductance a little over the expected 6 nS due to a small background conductance). First the voltage was stepped from +69 to −45 mV in order to close the two channel 2. A rapid drop in conductance (<47 ms) of ~3.4 nS was observed, consistent with about two channel 2 closures. Then the voltage was stepped back to +69 mV to close channel 3 before channel 2 could reopen. An immediate decrement of conductance from 3.3 to 0.5 nS was seen, indicating the closure of two channel 3. Soon after the drop, two increments, each of about 3 nS, were detected. This was interpreted to be two synchronized reopenings of channels 2 and 3. This recording is typical of many observations.

Strong cooperativity between channel 2 and 3 was evident in observations of simultaneous reopening. When channel 3 was closed, the reopening of 2 occurred virtually simultaneously with the reopening of channel 3. Figure 8 shows a record with three sequential voltage ramps showing virtual simultaneous reopening of channels 2 and 3. The shaded portion on the left side of panel a was converted to conductance (panel b), clearly showing the double-sized conductance increase. Panel c is an expanded view of that transition showing only one conductance change, within the limitation of the current digitization, consistent with simultaneous opening of 2 and 3. An additional demonstration of this cooperativity was performed using a voltage pulse sequence (Figure 9). The membrane contained two triplins, and in both, channel 1 had been closed (not shown). The voltage was first held at +69 mV where channels 2 and 3 are open. Then, the voltage was switched to −45 mV and both channel 2 closed almost immediately. Then, the voltage was returned to +69 mV. With insufficient time to reopen channel 2, both channel 3 closed almost immediately. Then a few hundred milliseconds later there are two double reopenings: channels 2 and 3 opening simultaneously. In this way, the conductance returned to the initial value.

The model also explains the slow closure of all three channels when a constant +70 mV potential was applied (Figure 1B). At constant high positive potential, the closure of channels 1 and 3 is favored by the applied voltage but slowed by interactions with channel 2. When both of these are closed, the strong cooperative effect allows channel 2 to close despite the fact that the applied voltage should favor channel 2 opening (Figure 1A). The conflicting actions of the electric field and the cooperativity result in a slow rate of closure indicated by the snail pathway as shown in Figure 7. The most conflicted channel would be channel 2 and indications of slower closing of channel 2 are present in Figure 1A,B. In Figure 1A, the third closing event is delayed, presumably channel 2. In Figure 1B, there are 3 delayed closings corresponding to the three triplins present.

### 2.4. Further Experimental Support for the Kinetic Model

**Figure 10:**
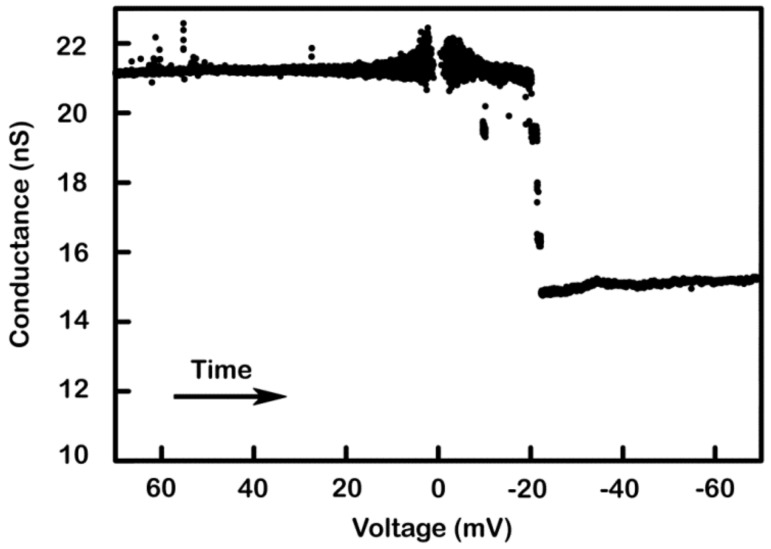
Supportive evidence of the model: multi-triplin containing membranes. A 2 mHz (0.28 mV/s) triangular voltage ramp (± 70 mV) was applied to a membrane. In the illustrated segment the voltage changed from positive to negative values and at −20 mV, four decrements are visible indicating the closure of four channel 2s. Of the remaining conductance, only 6 nS could be due to the remain channel 3s of the active triplins. The remaining nine nS (total of 15 nS) are consistent with the two non-gating triplins. Therefore, at positive voltages the conductance was composed of two non-gating triplins and four gating triplins i.e. with their channel 1s closed (3.0 nS per triplin) for a total of 21 nS.

Further evidence of the model comes from its ability to account for conductance changes observed in multi-triplin experiments. In Figure 10, the initial conductance was about 21nS. As the voltage ramp slowly changed the voltage from positive to negative values, one observed 4 conductance decrements at negative voltages. These must be four closures of channel 2. This indicates that four channel 1s (1.5 × 4 = 6 nS) must have been closed beforehand to allow the channel 2s to close. Therefore, the all-open conductance should have been 21 + 6 = 27 nS, equivalent to 6 triplins. The 21nS conductance before the four closing events must have been composed of two triplins with channel 1 open (9 nS) plus four triplins with channel 1 closed (12nS) for a total of 21 nS. (recall that the triplins do not respond to voltage until channel 1 is closed) The remaining conductance after the four channel 2 closures is consistent with four open channel 3s (4 × 1.5 nS) plus two triplins (2 × 4.5 nS) that were not gating resulting in a final conductance of 15 nS, essentially the observed value. Thus, the observed conductances and conductance changes in multi-triplin membranes can be understood based on the model.

**Figure 11:**
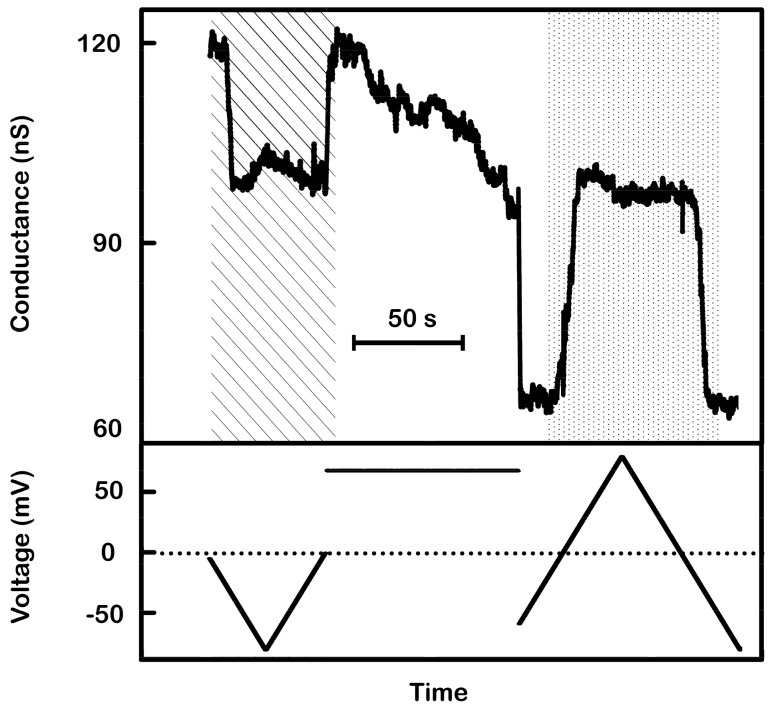
Supportive evidence of the model: activation of non-responding triplins by closing channel 1s. A 9 mHz (1.44 mV/s) triangular voltage ramp (± 80 mV) was applied to a membrane containing many triplins. As the voltage became more negative the conductance decreased rapidly by 17 nS, equivalent to the closure of 11 channel 2’s (each 1.5 nS) (in striped region). As the voltage moved toward zero, that same conductance increased once again indicating the reopening of those channels. Thus, only 11 triplins had a closed channel 1 and were capable of gating. The subsequent application of +70 mV for 85 s produced a conductance decrement of 24 nS, corresponding to the closure of 16 channel 1s. Reapplication of the voltage ramp (dotted region) showed conductance changes at negative potentials of 38 nS, equivalent to the closure and reopening of 25 to 27 channel 2’s.

How do we know that the non-gating triplins are really there? The answer is that some of the non-active triplins can be activated by closing the channel 1’s in the inactive triplins. In the experiment illustrated in Figure 11, the total initial conductance (120 nS) is consistent with about 20 non-gating triplins (all channels open) and 11 gating triplins (determined from the 17 nS drop due to channel 2 closures as the ramp voltage became negative). Notice how the channels reopened as the ramp moved toward positive values. Then, the applied voltage was switched from the ramp to a constant +70 mV potential. During the 85 s of application of the +70 mV potential, the conductance dropped. There were too many channels in the membrane to see individual conductance decrements, but the conductance declined by about 24 nS. That would correspond to 16 closures of channel 1 and thus the activation of 16 more triplins. Adding those to the original 11 would make 27 active triplins. The reapplication of the voltage ramp resulted in 38 nS drop in conductance at negative potentials. This corresponds to 25–27 closures of channel 2, consistent with 25–27 gating triplins. Thus, the non-gating triplins can be activated by the closure of channel 1. Again, the model explains the observed conductance changes.

### 2.5. Triplin Seems to Be Fundamentally Different from the Porins Described to Date

The fact that triplin is a three-channel unit and the channels have a relatively high conductance raises the possibility that triplin might be a porin. Perhaps the conditions used in analyzing the channels (membrane lipid composition, salt solutions, etc.) altered the properties of well-known bacterial channels, such as OmpC and OmpF. This possibility was tested by obtaining pure OmpC and OmpF from Mathias Winterhalter. These were reconstituted into planar membranes under identical conditions to those used for triplin and found to have the properties reported in the literature [15,16,17,18]. The possibility that triplin activity might be produced by maltoporin was tested by adding maltose, but no inhibition was detected. PhoE is a highly specific porin with anion selectivity [19,20]. Triplin shows cation preference (P_K_^+^/P_Cl_^−^ = 3.6 ± 0.3 (0.1M KCl vs. 1.0M KCl)). A multi-porin knock-out strain was also tested (Omp9), lacking OmpF, LamB, OmpA, OmpC, and OmpN, provided by Liviu Movileanu. This strain had normal triplin activity (Figure S2). Knowing the many reports of cryptic porins expressed when the major porins are knocked out, a search was undertaken for proteins with sequence homology to OmpF and OmpN. SWISS-MODEL from ExPASY was used to identify gene products that seemed to have the capacity to form channel-like structures. We identified the following: RhsD, RhsE, HtrE, and FlgE. Knock-outs in each of these were purchased from the Coli Genetic Stock Center. When tested, all of those had the triplin activity. Based on these studies, we believe that triplin is not related to the known porins.

## 3. Discussion

The unique and remarkable properties of the triplin channel complex indicate that it must be involved in some important but as yet undetermined physiological function in *E. coli*. The electrophysiological properties of triplin demonstrate that it consists of three conducting units with virtually identical conductance and ion selectivity. These conducting units, channels, exhibit both steep voltage dependence and a high degree of positive cooperativity. The channels prefer to be in the same state whether it be the closed or open state possibly due to the dipole-dipole interaction of their voltage sensor. Thus, the same structure could account for both properties. However, the cooperativity seems to be limited to pairs of likely adjacent channels: 1/2 and 2/3. The voltage-dependent closure of channels 1 and 3 at positive potentials and channel 2 at negative potentials is most simply explained by channel 2 being oriented in an opposite direction to 1 and 3. This opposite orientation is necessary for the cooperativity if it were based on the interaction between the charged dipoles of the voltage sensors on each channel. An opposite orientation of adjacent channels would result in a favorable dipole–dipole interaction (1/2 and 2/3) (Figure 7). The movement across the membrane of the voltage sensor upon channel closure would invert the dipole, resulting in electrostatic repulsion rather than attraction between adjacent channels and thus favor closure of the adjacent one (see Figure 7). This model fits the results most simply if the triplet were organized as a linear arrangement of channels, with channel 2 in between 1 and 3. In a linear arrangement, channel 1 would only interact with channel 2, and likewise for channel 3. This would fit well with the functional observations. In the model, channels 1 and 3 are essentially identical, and when either one closes at high applied positive potentials, the rapid, low voltage closure process begins with the closure of channel 2 at negative potentials. The opposite signs of the potentials required for gating, the steepness of the voltage dependence, and thus the requisite transmembrane movement of a large amount of gating charge in opposite directions cannot be easily explained by a process other than an opposite orientation for these channels, despite the obvious concerns on how that could take place. Typically, the channel’s structure or the cellular machinery that inserts the protein limits insertion in only one direction. For example, the large surface domain of α- hemolysin determines the direction of insertion [21]. However, VDAC channels have been shown to be able to insert into planar membranes in both directions [22,23]. In addition, protein toxins (colicins) translocate most of their mass to the opposite membrane surface when they insert into a phospholipid membrane [24], showing that the presence of an aqueous domain does not necessarily inhibit the insertion of a protein into a membrane. Finally, there is at least one example of a protein where the subunits have inverted transmembrane topology—EmrE [25]. Thus, there is no theoretical impediment to the possibility of subunits of channel-forming proteins inserting in opposite directions save for the need to be skeptical with regard to any novel proposal. 

A Boltzmann distribution analysis of the voltage dependent conductance of channel 2 yielded an estimate of the effective number of gating charges that must translocate through the entire transmembrane electrical potential difference in order to account for the observed voltage dependence. The value of 14 that was obtained for triplin is twice that reported for the voltage gated channels responsible for electrical excitability in animal cells when channel activation was analyzed using the Boltzmann distribution [1,26]. When the amount of charge translocated was estimated by measuring displacement currents [7,26,27], values comparable to those reported here were obtained. Thus, by comparison, one can conclude that the steepness of voltage gating of triplin channels is extremely high indicating an as yet undefined but important role in the physiology of *E. coli*.

The very steep voltage dependence requires a highly charged voltage sensor domain. Positively charged helices have been shown to be the voltage sensors in the Na^+^/K^+^/Ca^2+^ family of voltage-gated channels [28]. However, the high conductance and triplet nature of this channel-former indicate that triplin is not part of the classical family of voltage-gated channels.

## 4. Materials and Methods 

Phospholipids were obtained from Avanti Polar Lipids (Alabaster, AL). Cholesterol was purchased from Sigma (St. Louis, MO, U.S.A.). A multi-porin knock-out strain, Omp9, was a generous gift by Liviu Movileanu. Omp9, *E. coli* BL21 (DE3) omp9 (F- hsdSB (rB- mB-) gal ompT dcm (DE3) ΔlamB ompF::Tn5 ΔompA ΔompC ompN::Ω, lacks OmpF, LamB, OmpA, OmpC, and OmpN. Strains of *E. coli* with the following proteins knocked-out: RhsD, RhsE, HtrE, and FlgE, were purchased from the Coli Genetic Stock Center (http://cgsc.biology.yale.edu/). Samples of pure OmpC and OmpF were generous gifts of Mathias Winterhalter.

### 4.1. Preparation of Channel-Forming Extracts from E. coli

DH5α *E. coli* cells were grown in 2 L of Terrific Broth Medium (TBM) to an O.D.600nm = 0.8 to 1. The cells were pelleted at 3000 g for 15 min (Beckman (Indianapolis, IN, U.S.A.) J-6B centrifuge) and the pellets resuspended in 30 mL of TEN* buffer (20 mM Tris-HCl, 5 mM EDTA, 500 mM NaCl, pH 8.0) prior to overnight storage at −80 °C. All subsequent steps were performed on ice or at 4 °C. The cells were lysed using a French press at 1000 psi (two passes). The lysate was centrifuged at 40k RPM (50Ti rotor) for 30 min at 4 °C, and the supernatant passed through a 0.2 µm filter prior to applying it to a 10 mL chitin column (New England Biolabs, Ipswich, MA, U.S.A.) preequilibrated with TEN buffer (20 mM Tris-HCl, 1 mM EDTA, 500 mM NaCl, pH 8.0). The application of the sample to the column must be slow (about 0.5–1 mL/min). The column was washed with 50 mL of TEN buffer, and then 10 mL of TEN + 30 mM DTT was allowed to flow through the column leaving 1 mL on the surface. After three days, the sample was washed off the column with 15 mL of TEN buffer. The sample was dialyzed overnight (12 kDa cut-off) against 3 L of 10 mM Tris-HCl, 1 mM EDTA, pH 8.0, and then for another 24 h against 5 L of 10 mM Tris-HCl pH 8.0. The sample was then filter-sterilized, supplemented with glycerol to 10% (*v*/*v*), flash frozen in 100 µL aliquots in thin-walled glass tubes, and stored at −80 °C. Samples were thawed only once and never re-frozen.

### 4.2. Electrophysiological Recordings

Planar phospholipid membranes were formed by the monolayer method [13,14]. The monolayers were formed by layering the lipid solution (0.5% (*w*/*v*) DPyPC, 0.5% (*w*/*v*) asolectin (polar extract of soybean phospholipids), and 0.05% (*w*/*v*) cholesterol in hexane) on the surface of the aqueous solutions (1.0 M KCl, 1 mM MgCl_2_, and 5 mM PIPES, pH 6.9) on either side of the partition. The membrane was formed across a 0.1 mm hole in the partition. Calomel electrodes were used to interface with the aqueous phase. The membrane voltage was clamped using a high-quality operational amplifier in the inverted mode and the current recorded using Clampex 10.3 software. Data was generally low-pass filtered at 500 Hz, but in some experiments, this was reduced to 5 kHz. The freshly thawed samples were supplemented with octyl glucoside to a final concentration of 1% (*w*/*v*), and 15–30 µL was dispersed in 5mL of aqueous solution on one side of the membrane. Voltage ramps were applied to investigate the behavior of these channels and determine their voltage-gating parameters. Triplin channels were observed and studied in over 100 separate experiments performed over a six-year period by 14 investigators.

### 4.3. Statistical Analysis

Aggregate results are reported as an average ± one standard deviation with the number of independent experiments indicated in parentheses. The symbol, N, is used to indicate the number of experiments performed with a specific protocol. Unless otherwise specified, the probability to observe the behaviors reported here when we tested the protocols is 100%. Significant differences in values were tested using the Student’s *t*-test.

### 4.4. Theory and Calculations: Quantification of Voltage Dependence

The Boltzmann distribution was used to determine the parameters of voltage dependence.

$$\frac{P_{O}}{P_{C}}=e^{-\frac{\Delta E}{RT}}.$$

$$\Delta E=nFV_{0}-nFV=nF\left(V-V_{0}\right)$$

$$\ln(\frac{G_{max}-G}{G-G_{min}})=\frac{nF}{RT}\left(V-V_{0}\right),$$

where *P_O_* and *P_C_* are the probability of the channels being in the open and closed state, respectively. Δ*E* is the energy difference between open and closed state. *nFV*_0_ is the energy difference in the absence of a membrane potential, whereas *nFV* is the voltage-dependent energy difference. *n* is the number of charges that would need to move across the entire electric field to account for the voltage dependence, and *V*_0_ is the voltage at which half of the channels on the membrane are open. *G*, *G_max_*, and *G_min_* are the conductance at any voltage, the maximal conductance, and the minimal conductance, respectively. RT is thermal energy. Important conditions for using the Boltzmann distribution are (1) the channel only has two states, open and closed, and (2) the distribution is determined at equilibrium. When using voltage ramps, the rate of change in voltage may be too fast to allow the system to reach equilibrium. Therefore, we determined the value of “n” at various rates of voltage change and extrapolated the fitted line of the n vs. rate plot to zero rate in order to estimate the value of “n” at equilibrium. For single-triplin experiments, we averaged the current records from at least 20 consecutive triangular ramps, and for multi-triplin experiments, we averaged the records from at least five ramps to obtain valid parameters. The use of voltage steps to collect data for conductance voltage curves is problematic for large channels because keeping the channel at a fixed voltage for some time results in time-dependent adaptation to the voltage that differs from one voltage level to the next, degrading the information obtained.

## 5. Conclusions

The triplin channel complex is unlike any membrane channel described to date. The very steep voltage dependence, the high cooperativity, the domino-like behavior of the three-channel unit, all are properties of a sophisticated molecular machine that has evolved to perform some important function in *E. coli*. These properties do not seem to resonate with any known function of these bacteria, thus hinting at the existence of as yet undefined physiological functions. From a biophysical standpoint, the mechanistic basis for the properties described is intriguing and worth exploring.

## Supplementary Materials

Supplementary materials can be found at https://www.mdpi.com/1422-0067/20/18/4501/s1.

## Abbreviations

DTT	Dithiothreitol
EDTA	ethylenediaminetetraaceticacid
PIPES	piperazine-N,N′-bis(2-ethanesulfonic acid)
Tris	trishydroxymethylaminomethane
DPyPC	Diphytanoylphosphatidylcholine
RPM	Revolutions per minute
