# Supplementary material for: Cooperativity and Steep Voltage Dependence in a Bacterial Channel
Source: Int J Mol Sci. 2019 Sep 11;20(18):4501. doi: 10.3390/ijms20184501 (PMC6770917; doi:10.3390/ijms20184501)
Supplement: Supplementary file 1 [file ijms-20-04501-s001.pdf]

Supplemental Material

When triplin is in the fully open state, i.e. when channels are open, channel closure is only observed when a positive transmembrane potential is applied, positive on the side of triplin addition. The application of a negative potential does not result in a cascade of channel closing events. The supplemental Figure 1 illustrates this. In addition to showing that triplin itself is asymmetric, this also shows that all the triplins inserted into the membrane with the same orientation.

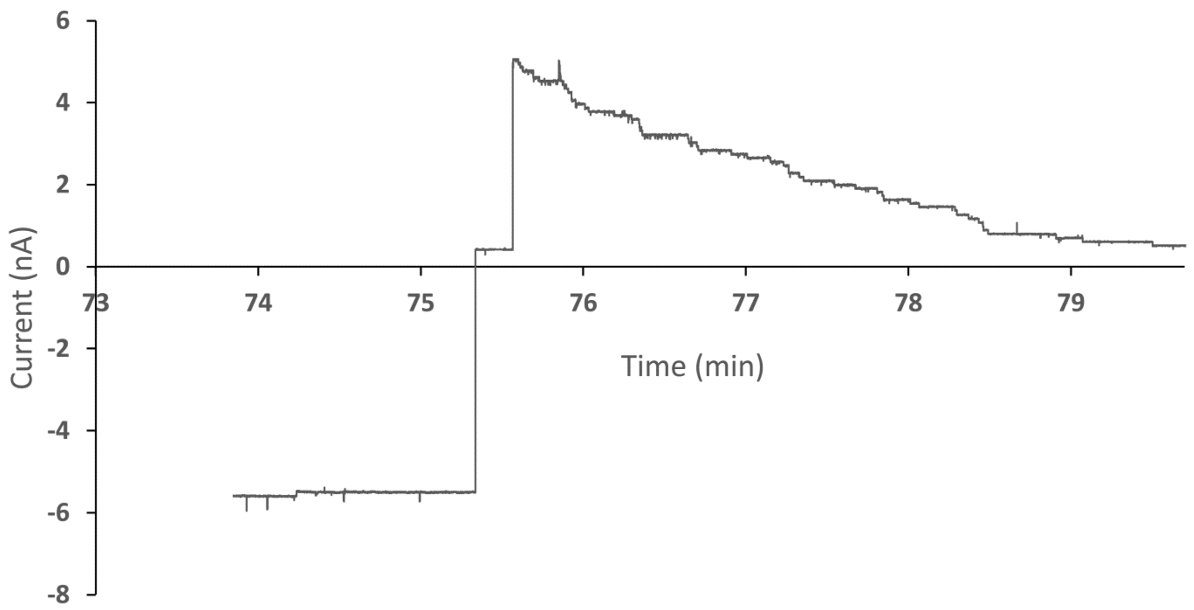

Figure 1 supplemental. Asymmetrical gating of triplin. The membrane contained many triplin channels. In the segment of the current recording shown, -73 mV was applied to the membrane for about 2 minutes followed by a change to +6 mV and finally the application of +66 mV.

With regard to the protein responsible for the triplin activity, Omp 9, a strain of *E. coli* with many porins knocked out, was tested to determine if this strain still produced triplin. It did and a critical property of triplin, the voltage gating of channel 2 was quantitated. The results are illustrated in supplemental Figure 2. The value obtained for the effective charge on the voltage sensor was 10.6. This is typical of the value determined for triplin obtained from wild-type *E. coli*.

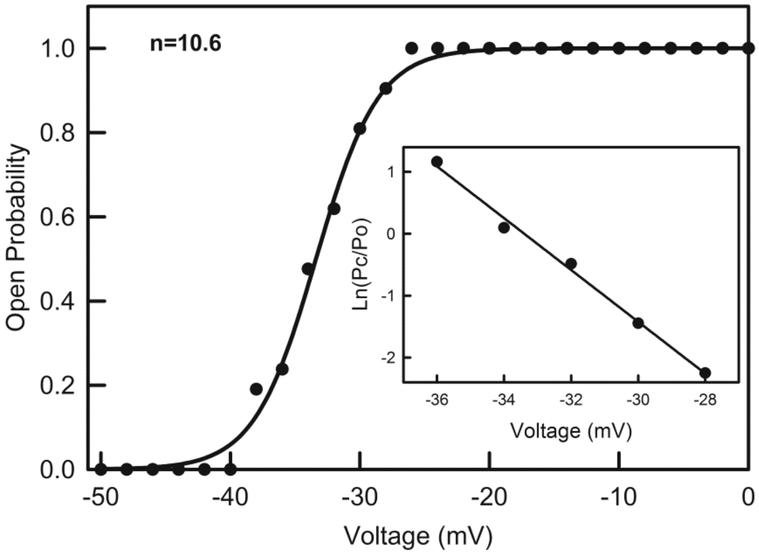

Figure 2 supplemental. Voltage gating of triplin channel 2 isolated from Omp 9. A single triplin formed in a planar membrane and after channel 1 was closed, voltage gating of channel 2 was observed for many ramps (30 mHz, 10.4 mV/sec). The probability of channel 2 being open as a function of voltage was determined (main figure). That probability was fitted to the Boltzmann distribution and the log to the base e transform plotted in the inset. The effective number of charges on the voltage sensor was calculated to be 10.6.
